# Cytosolic diffusivity and microscopic anisotropy of *N*‐acetyl aspartate in human white matter with diffusion‐weighted MRS at 7 T

**DOI:** 10.1002/nbm.4304

**Published:** 2020-03-31

**Authors:** Henrik Lundell, Carson Ingo, Tim B. Dyrby, Itamar Ronen

**Affiliations:** ^1^ Danish Research Centre for Magnetic Resonance, Centre for Functional and Diagnostic Imaging and Research Copenhagen University Hospital Hvidovre Denmark; ^2^ Department of Physical Therapy and Human Movement Sciences Northwestern University Chicago Illinois; ^3^ Department of Neurology Northwestern University Chicago Illinois; ^4^ Department of Applied Mathematics and Computer Science Technical University of Denmark Kongens Lyngby Denmark; ^5^ C. J. Gorter Center for High Field MRI, Department of Radiology Leiden University Medical Center Leiden The Netherlands

**Keywords:** cell‐specific morphology, diffusion, human brain, intra‐axonal space, microscopic anisotropy

## Abstract

Metabolite diffusion measurable in humans in vivo with diffusion‐weighted spectroscopy (DW‐MRS) provides a window into the intracellular morphology and state of specific cell types. Anisotropic diffusion in white matter is governed by the microscopic properties of the individual cell types and their structural units (axons, soma, dendrites). However, anisotropy is also markedly affected by the macroscopic orientational distribution over the imaging voxel, particularly in DW‐MRS, where the dimensions of the volume of interest (VOI) are much larger than those typically used in diffusion‐weighted imaging. One way to address the confound of macroscopic structural features is to average the measurements acquired with uniformly distributed gradient directions to mimic a situation where fibers present in the VOI are orientationally uniformly distributed. This situation allows the extraction of relevant microstructural features such as transverse and longitudinal diffusivities within axons and the related microscopic fractional anisotropy. We present human DW‐MRS data acquired at 7 T in two different white matter regions, processed and analyzed as described above, and find that intra‐axonal diffusion of the neuronal metabolite *N*‐acetyl aspartate is in good correspondence to simple model interpretations, such as multi‐Gaussian diffusion from disperse fibers where the transverse diffusivity can be neglected. We also discuss the implications of our approach for current and future applications of DW‐MRS for cell‐specific measurements.

## Graphical abstract

Metabolite diffusion measurable in humans in vivo with diffusion‐weighted spectroscopy provides a window into the intracellular morphology and state of specific cell types. Anisotropic diffusion in white matter is markedly affected by the macroscopic orientational distribution over the imaging voxel. We suggest a method for discarding any rotational order, which provides robust quantification across brain regions with different degrees of alignment.

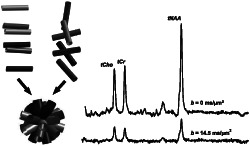

## 1 INTRODUCTION

The anisotropic mobility of water molecules observed with diffusion‐weighted imaging (DWI) is a sensitive and noninvasive in vivo biomarker for changes in tissue microstructure and microscopic organization, but is nonspecific, as water is present in all extra‐ and intracellular spaces and exchanges across different compartmental environments.^1^, ^2^, ^3^ To resolve the ambiguous sources of information in heterogeneous tissues, diffusion‐weighted spectroscopy (DW‐MRS) was introduced to separate the water signal from that of the less abundant metabolites, which only reside in the intracellular environments of specific cell types.^4^, ^5^, ^6^ In the central nervous system, *N*‐acetyl aspartate (NAA) and glutamate (Glu) reside mainly in neuronal cell bodies, dendrites, and axons.^7^ The contributions from extracellular environments for these metabolites are presumably negligible. DW‐MRS has been successfully applied to both animals and humans since the early 1990s,^8^, ^9^ and has been applied to show cell‐specific alterations in cerebral stroke, tumors, healthy aging, multiple sclerosis, and systemic lupus erythematosus.^10^, ^11^, ^12^, ^13^, ^14^, ^15^, ^16^ Besides its use as a cell‐specific biomarker, DW‐MRS can also provide input regarding the contributions from individual cell spaces in biophysical interpretations of water diffusion data.^17^

The interpretation of the DW‐MRS data in terms of the microscopic cell structure is confounded by the entangled contributions from anisotropic structures with different orientations at a subvoxel level. When considering white matter axons, the effects of, eg, crossing, bending, and disperse axons modulate the global anisotropy within the measurement volume of interest (VOI) and introduce the nonmonoexponential attenuation of the diffusion‐weighted signal concerning the *b‐*value.^18^, ^19^, ^20^ The dispersion, thus, affects simple diffusion metrics, obtained from, eg, the diffusion tensor,^21^ such as the fractional anisotropy (FA), mean diffusivity (MD) and transverse and longitudinal diffusivities (*D*_T_ and *D*_L_), making them less informative as microstructural markers in the context of such large acquisition volumes as those used in DW‐MRS. Importantly, these metrics are often calculated by relying on a monoexponential attenuation, which will also depend on the *b‐*value. Two types of fiber organization require no estimation of the orientational distribution: subdomains in which fibers are either perfectly aligned or fully dispersed. The latter case is closer to a realistic scenario encountered in DW‐MRS experiments. Kroenke et al pioneered this view and showed that the nonmonoexponential decay of NAA in the rat brain could be well described by uniformly distributed “sticks,” ie Gaussian diffusion tensors with zero transverse diffusivity.^22^ Revisiting the same model, Palombo et al recently performed a similar DW‐MRS experiment in the mouse brain with a similar conclusion for the intraneuronal diffusivity.^23^

In contrast to rodent models, where gray matter with highly dispersed dendritic fibers dominates at the typical voxel resolution, DW‐MRS in humans allows for experiments on well defined volumes in either gray or white matter, revealing significant differences in the metabolite diffusion properties across tissue types.^24^, ^25^, ^26^ It has still not been investigated how the findings on rodents mentioned above translate to human white matter. Obtaining microstructural information from the DW‐MRS measurements performed in large volumes can be done using two different approaches, inspired by the field of DWI. One approach is to fit a model of the macroscopic orientational distribution obtained from the high‐resolution diffusion tensor imaging (DTI) water data acquired with several gradient directions.^17^, ^23^, ^27^ This view has allowed for the simultaneous quantification of the angular dispersion of the axons and isolation of the cytosolic diffusivity of NAA but also highlights that orientational dispersion also affects high‐resolution water DTI data.^28^ The second approach, applied and discussed in this study, discards the information regarding the macroscopic organization by averaging the DW‐MRS data acquired with uniformly distributed gradient directions. This approach, referred to in the literature as *powder averaging* or *spherical averaging*, generates data that mimic a perfectly uniform orientational distribution. The term “powder averaging” is borrowed from solid‐state NMR, where it refers to spectra acquired from powdered solid‐state samples, to allow an orientationally uniform representation of, eg, a chemical shift or a dipole‐dipole coupling that is anisotropic with respect to the *B*_0_‐field orientation.^29^ The term has recently been adopted by the DWI community, and the method of averaging diffusion‐weighted data from evenly distributed gradient orientations was proposed as a simple way to handle unknown fiber configurations.^30^, ^31^, ^32^, ^33^, ^34^

In this paper, we suggest the use of powder averaging as simple and robust approach to acquire and analyze human DW‐MRS data. We combine for human DW‐MRS contexts large diffusion weightings with high angular resolution and demonstrate comparable diffusion metrics from tNAA in white matter derived from the DW‐MRS data acquired in two white matter regions with widely different degrees of axonal dispersion. We discuss simple and experimentally feasible interpretations of local intracellular diffusivity and evaluate the experimental parameter choice and error propagation in simulations.

## 2 METHODS

### 2.1 Human subjects

Ten healthy participants (28.5 ± 9.5 years old, five males and five females) participated in the study. The study followed the guidelines of the Leiden University Medical Center Institutional Review Board, and informed consent was obtained from all subjects before the experiments.

### 2.2 MRI and DW‐MRS experiments

**FIGURE 1:**
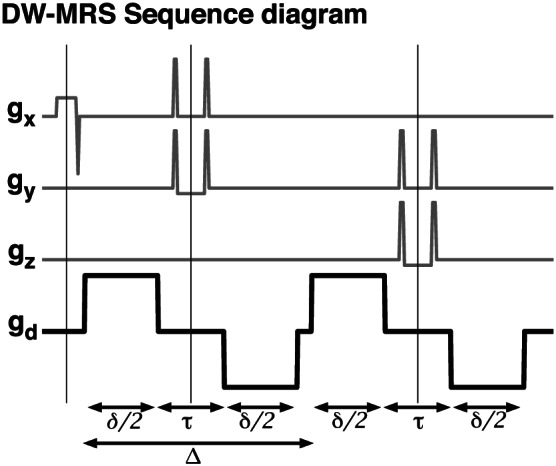
Sequence diagram of the DW‐MRS sequence used in the study. Thin vertical lines indicate excitation and refocusing pulses. The gradients for spatial PRESS localization are shown in gray, and the diffusion‐encoding gradient (applied in varying directions) is in black. Encoding gradient duration (*δ*) and separation (*Δ*), and bipolar delays (*τ*), are indicated

The measurements were performed on a 7 T human MRI system (Philips Healthcare, Best, The Netherlands) using two‐channel transmit and 32‐channel receive coils (Nova Medical, Wilmington, Massachusetts). All participants were scanned with a short survey scan followed by a short 3D *T*_1_‐weighted gradient‐echo sequence with 1 × 1 × 1 mm^3^ resolution and *T*_R_/*T*_E_ = 4.9/2.2 ms. The participants were then either scanned with a VOI placed in the mid‐sagittal plane over the anterior body of the corpus callosum (CC, *N* = 5) or in left parietal white matter (PWM, *N* = 5). VOI placement was planned on the *T*_1_‐weighted image with NAA as reference metabolite. Bipolar diffusion‐weighted gradients were incorporated in a point‐resolved spectroscopy (PRESS) sequence^17^ as shown in Figure 1 with the relevant timing parameter defined. The following parameters were used for the CC VOI: *T*_E_ = 125 ms, VOI size 25 × 15 × 10 mm^3^. The diffusion weighting was achieved with bipolar gradient pairs around the two inversion pulses with total encoding gradient pulse duration *δ* = 45 ms distributed around each inversion pulse with a bipolar delay *τ* = 14 ms and a gradient separation of *Δ* = 59 ms. Five *b*‐values in the range 0‐9.4 ms/μm^2^ were achieved with gradient amplitudes 0, 10, 20, 30, and 40 mT/m. Parameters used for the PWM VOI were *T*_E_ = 136 ms, VOI size 16 × 16 × 16 mm^3^. To achieve sufficient *B*_1_ in the PWM VOI an additional high‐permittivity dielectric pad was placed between the participants' heads and the receive coil as previously described.^26^ Diffusion weighting for the PWM VOI was performed as for the CC VOI but with *δ* = 53 ms, *Δ* = 65 ms, and *τ* = 12 ms, resulting in *b‐*values in the range 0‐14.5 ms/μm^2^. The *b*‐values were calculated from the diffusion‐weighting gradients only. The additional contributions from the crusher and slice gradients were 0.0034 ms/μm^2^ without diffusion‐encoding gradients calculated from the trace of the diffusion‐weighting *b*‐tensor. The effects of cross‐terms deviated maximally at the intended 14.5 ms/μm^2^ for the PWM acquisition and were in the range of ±0.07 ms/μm^2^ depending on gradient direction. This fact was neglected in the following analyses, but the possible bias is assessed in the supplementary material (see Figure S1). The 12 uniformly distributed gradient directions used were constructed from an electrostatic repulsion simulation^29^ and repeated with 12 repetitions for each diffusion‐weighting condition divided into four phase cycles. The water suppression was adjusted to maintain a sufficient residual water peak for the post hoc phase, and frequency drift corrections with an amplitude 5‐10 times higher than the NAA peak for all diffusion conditions, as previously described.^35^ Higher axonal alignment in the CC compared with PWM results in more signal attenuation for directions parallel to the fiber bundle (see Figure 3 later), limiting the maximum *b*‐value for the CC to a value lower than the one used for the PWM. A peripheral pulse unit triggered acquisitions with the *T*_R_ set to three heartbeats. Additional reference data for the eddy current corrections were acquired for each diffusion‐weighting condition without water suppression with four repetitions per condition. The total scan time was approximately 48 min for the DW‐MRS scan including water references.

**TABLE 1:** Fitted diffusion parameters from powder‐averaged data in the CC and PWM with longitudinal and transverse diffusivities (*D*_L_ and *D*_T_), derived MD and μFA from Equation 1 and *D*_L_(stick) from Equation 2

	CC, mean (sdev)	PWM, mean (sdev)	*p*‐value
*D*_L_	0.51 (0.1) μm^2^/ms	0.50 (0.07) μm^2^/ms	0.83
*D*_T_	0.024 (0.01) μm^2^/ms	0.011 (0.01) μm^2^/ms	0.13
MD	0.19 (0.04) μm^2^/ms	0.17 (0.02) μm^2^/ms	0.51
μFA	0.94 (0.05)	0.98 (0.03)	0.25
*D*_L_(stick)	0.65 (0.1) μm^2^/ms	0.58 (0.08) μm^2^/ms	0.29

### 2.3 Spectral preprocessing and quantification

The individual spectra were corrected for eddy currents and zero‐order phase and frequency variations with the residual water peak as reference for each condition, as described earlier using in house software written in MATLAB (MathWorks, Natick, Massachusetts).^25^ The corrections were performed in the frequency domain, and the corrected data were retransformed to the time domain for later processing. The metabolite signal levels and Cramér‐Rao lower bound (CRLB) noise estimates for tNAA (NAA + NAAG) were quantified for each *b*‐value and direction using LCModel^36^ with an appropriate simulated basis set. Signal to noise ratio (SNR) was estimated as the ratio between the mean and standard deviation of the *S*(*b* = 0) signals acquired with 12 repetitions. Powder averaging was performed over gradient directions for each unique *b*‐value.

#### 2.3.1 Diffusion models

For an ensemble of uniformly distributed and nonexchanging domains described by monodisperse diffusion tensors (such as those associated with diffusion in thin fibers) with the unweighted signal *S*_0_ and longitudinal and transverse eigenvalues *D*_L_ and *D*_T_, the signal attenuation is given by

(1) $$S\left(b\right)=S_{0}\;e^{-bD_{T}}\frac{\sqrt{\pi }}{2}\frac{\begin{array}{c} \;\\ \mathrm{erf}\left(\sqrt{b\left(D_{L}-D_{T}\right)}\right) \end{array}}{\sqrt{b\left(D_{L}-D_{T}\right)}}$$

where *S*_0_ is the non‐diffusion‐weighted signal. Equation 1 was first proposed by Callaghan et al in a study of anisotropic diffusion in wheat grain endosperm.^37^ The view of diffusion as the ensemble average of rotationally disperse anisotropic components gives an important understanding of diffusion‐weighted data. Equation 1 was first applied for neuronal tissue by Kroenke et al and used for the interpretation of DW‐MRS data with low angular resolution assuming a highly disperse sample.^22^ Assuming diffusion in cylindrical geometries with negligible radius, one can assume *D*_T_ = 0, and Equation 1 reduces to the disperse “stick” model:

(2) $$S\left(b\right)=S_{0}\frac{\sqrt{\pi }}{2}\frac{\mathrm{erf}\left(\sqrt{bD_{L}}\right)}{\sqrt{bD_{L}}}.$$

Equations 1 and 2, referred to as “tensor” and “stick,” were fitted to the experimental data, including the *S*(*b* = 0) datapoint, using nonlinear least squares with *S*_0_ and diffusivities as fitting parameters. The fit was initialized by a linear fit of the MD and setting *D*_L_ = MD/3 and *D*_T_ = 0. Diffusivities were constrained to nonnegative values and nonplanar anisotropies (*D*_L_ ≥ *D*_T_ > 0). The microscopic fractional anisotropy (μFA), equivalent to the local FA of the individual subdomains (fibers) unaffected by the orientational distribution,^33^, ^38^ can then be derived as

(3) $$\mathrm{\mu FA}=\sqrt{\frac{{\left(D_{L}-D_{T}\right)}^{2}}{D_{L}^{2}+2D_{T}^{2}}}.$$

The compartment MD was calculated as (*D*_L_ *+ 2D*_T_)/3 for fits to Equation 1 or MD = *D*_L_/3 for Equation 2, reflecting the monoexponential initial slope of the two models. A two‐tailed *t*‐test was performed to examine if estimates from the two regions were significantly different, with a threshold set to *p* < 0.05. The systems well described by Equation 2 approach a characteristic $1/\sqrt{bD_{L}}$ asymptote for high *b* as the term $\mathrm{erf}\left(\sqrt{bD_{L}}\right)$ quickly approaches unity.^34^ In addition to the parameter estimates, we thus investigated the asymptotic behavior of the high‐*b‐*value data by comparing it with a linear scaling of *b* ^−1/2^. Given the stick model, the maximum sensitivity in signal for variation in *D*_L_ is found from the derivative of Equation 2 at *bD*_L_ = 3*b* MD = 2.285.

In addition to the microscopic compartment diffusivities described above, we also estimated the macroscopic diffusion tensor representing the disperse compartment averaged diffusion process. The directional diffusivities *D*_*i*_ was first estimated from the initial slope of the individual attenuation curves of the 12 gradient directions (row unit vectors **e**_*i*_) using a gamma distribution of diffusivities to account for the nonmonoexponential attenuation with respect to the *b*‐value.^33^ The voxel‐averaged diffusion tensor 〈**D**〉 was then found by solving the system of linear equations *D*_*i*_= $e_{i}\langle D\rangle e_{i}^{T}$ . The related 〈MD〉, 〈*D*_L_〉, 〈*D*_T_〉 and FA were found from the eigenvalues of 〈**D**〉**.** ^38^ With known microscopic diffusion tensors, the difference between the macroscopic averages 〈*D*_L_〉 and 〈*D*_T_〉 relates to the angular dispersion. We estimated *θ*, the angular spread from the tensor's symmetry axis, assuming disperse sticks with *D*_L_ = 3〈MD〉 from Equations 18 and 19 of Lasič et al.^33^

#### 2.3.2 Simulations

Synthetic data were produced to evaluate (i) the effect of the SNR in parameter estimates for varying underlying anisotropy and maximum *b*‐value (*b*_max_), (ii) the number of gradient directions (*N*_dir_) used for the powder average, and (iii) the interpretation of the transverse diffusivity in terms of cylindrical restrictions of different diameters. The mean error (ME) and the coefficient of variation (CoV) were evaluated for the different settings.

### 2.4 Noise propagation

We assumed substrates with varying μFA by adjusting *D*_L_ and *D*_T_ (following Equation 3) at a constant MD to consider similar levels of signal attenuation at the same *b*‐value. The synthetic signals were calculated using Equation 1 and the diffusion parameters were estimated from 10^4^ realizations of the data points computed over 12 averages with added noise drawn from a zero‐mean Gaussian distribution reflecting the noise characteristics of the complex averaged DW‐MRS data. The standard deviation of the noise was assumed to be constant over *b*‐values and set to 1/SNR relative to the SNR of the *b* = 0 measurements. As in the experimental data, a set of five *b‐*values from linearly spaced gradient amplitudes was used. Moreover, (MD *b*_max_) was scaled in the range between 0.1 and 5 to evaluate the effect of increasing maximum *b*‐value.

### 2.5 Number of directions

The rotational variance of a powder average is expected to increase with the underlying alignment of domains and their anisotropy, similarly to the previous observations of DTI parameters in numerical analysis.^39^ The greatest variance occurs in aligned subdomains equivalent to a single diffusion tensor, as in eg a highly organized white‐matter tract. Considering the rotation **R** of a cylindrically symmetric diffusion tensor **D**, the powder‐averaged *b*‐dependent signal from a set of *N =* [3, 6, 12, 24] uniformly distributed gradient directions described by the unit vectors **e**_*i*_ is given by

(4) $$S_{j}\left(b\right)=\frac{1}{N}\sum_{i=1}^{N}\exp\left(-be_{i}R_{j}DR_{j}^{T}e_{i}^{T}\right).$$

Equations 1 and 2 were fitted to the individual powder‐averaged signals computed from Equation 4 for a cylindrically symmetric diffusion tensor with diagonal elements [*D*_L_ *D*_T_ *D*_T_]. The standard deviations over 1024 uniformly distributed rotations **R** of the sample were estimated for each increment of gradient directions *N* used for the powder average. The rotations **R** were uniform with respect to the symmetry axis with the eigenvalue *D*_L_.

### 2.6 Restriction size

Monte Carlo simulations were performed using the diffusion gradient waveforms used in the experiments extracted from a sequence simulator in the MRI system's sequence programming environment (Paradise, Philips Healthcare, Best, The Netherlands). These simulations were performed to evaluate the effect of finite radii on the transverse diffusivity with the diffusion‐encoding gradients used in the experiments. Random walk processes were performed with 10^4^ walkers and a time step of 6.4 μs inside the cylindrical restrictions of varying radius and intrinsic free diffusion coefficient *D*_0_ using in‐house software written in MATLAB.

## 3 RESULTS

### 3.1 Experiments

**FIGURE 2:**
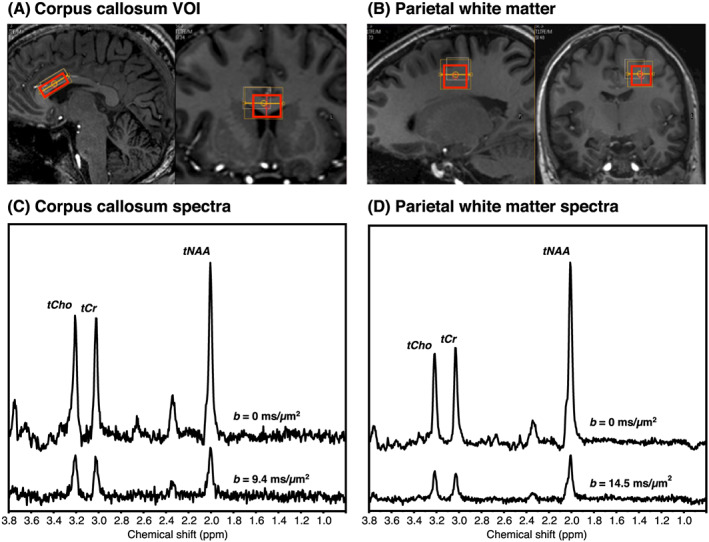
A, B, The VOI placement was planned on NAA (red box) in CC (A) and PWM (B) overlaid on a *T*_1_‐weighted image. C, D, Representative spectra from individual participants in CC (C) and PWM (D). Spectra are powder‐averaged over 12 directions and shown for *b* = 0 (upper spectra) and the highest *b*‐values obtained in the two regions (lower spectra). Baselines are shifted for visualization

**FIGURE 3:**
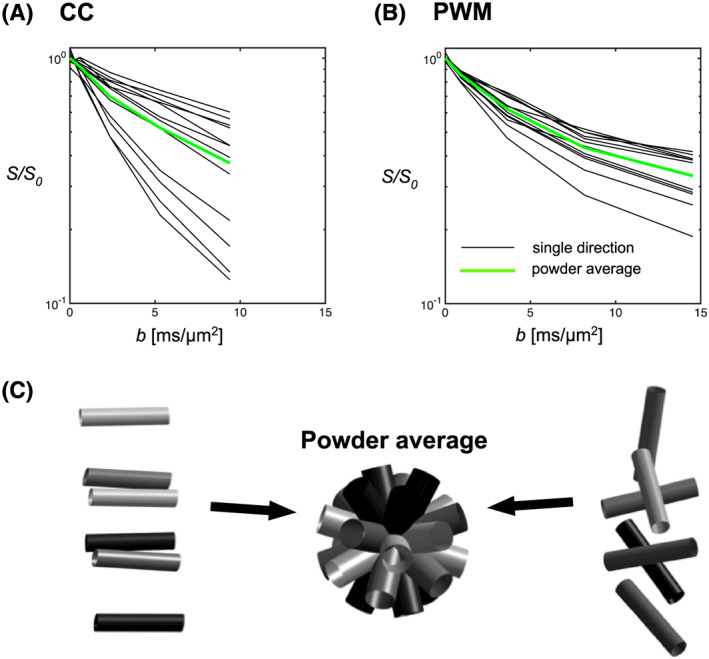
A, B, The attenuation curves from two individual subjects in the CC (A) and PWM (B) for the intraneuronal metabolite tNAA. Data are shown for measurements in 12 uniformly distributed gradient directions (black lines) and the powder average (gray‐green lines). C, Illustration of a region with fairly aligned axons as in CC (far left) and more disperse as in PWM (far right). The powder average (middle) represents the signal contribution from each original orientation as a uniformly distributed sample

**FIGURE 4:**
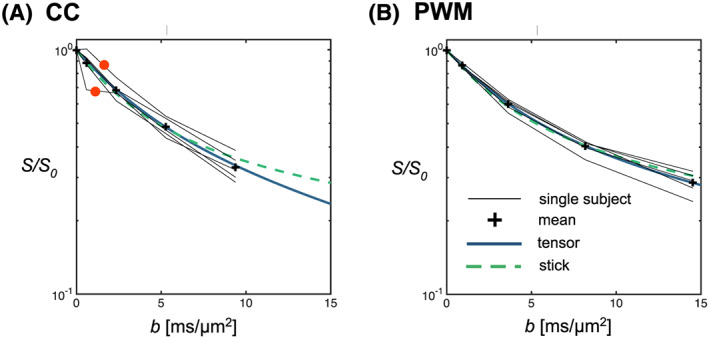
The powder‐averaged attenuation curves for tNAA in the CC (A) and PWM (B). The models of uniformly distributed cylindrically symmetric tensors and sticks are here fitted to the average of all participants. The two subjects in the CC acquisitions with apparent outliers are highlighted with red dots

Figure 2A and 2B shows the VOI placement and representative spectra for the lowest and highest *b*‐values in CC and PWM. CRLBs were in the range 1‐2% at *b* = 0 and 2‐3% for *b* = 14.5 ms/μm^2^ for tNAA in PWM and 2% at *b* = 0 and 3‐5% for *b* = 9.4 ms/μm^2^ in CC. SNR calculated from the repeated *S*(*b* = 0) signals were in the range 31‐67 in PWM and 21‐33 in CC. The lower noise figures in the PWM data are also clearly seen in the individual spectra in Figure 2C and 2D. The DW‐MRS data before and after powder averaging are shown in Figure 3. The larger degree of axonal alignment and, thereby, a higher *macroscopic* diffusion anisotropy in CC compared with PWM is reflected by the larger directional dependence in data, before powder averaging. Figure 4 shows the individual powder‐averaged attenuation curves from the individual subjects normalized to the estimated *S*_0_ from Equation 1 and the mean normalized signal for all subjects with fits for the tensor and the stick models. Fitted *S*_0_ was in good agreement with the experimental *S*(*b =* 0) data points (mean *S*(*b =* 0)/*S*_0_ was 1.003 and standard deviation 0.032).

The group averaged data are shown in Table 1, where MD = (*D*_L_/3 + 2*D*_T_/3) and μFA is the dispersion free microscopic fractional anisotropy calculated from *D*_L_ and *D*_T_. No significant difference in the parameter estimates were found between the two regions. Estimates from the macroscopic diffusion tensor are shown in Table 2. Estimates related to anisotropy (〈*D*_L_〉, 〈*D*_T_〉 and FA) are significantly different, which is also illustrated by a larger angular dispersion estimate (*θ*) from the PWM voxels. However, the rotationally invariant 〈MD〉 values re similar across the two regions and in good agreement with the estimates from the powder average.

**TABLE 2:** Data derived from the macroscopic VOI‐averaged diffusion tensor in the CC and PWM with longitudinal and transverse diffusivities (〈**D**_L_〉 and 〈**D**_T_〉), derived MD (〈**MD**〉), macroscopic FA and the estimated angular spread (*θ*) assuming monodisperse sticks with longitudinal diffusivity **D**_L_**=** 3〈**MD**〉

	CC, mean (sdev)	PWM, mean (sdev)	*p*‐value
〈*D*_L_〉	0.42 (0.07) μm^2^/ms	0.29 (0.05) μm^2^/ms	0.008
〈*D*_T_〉	0.080 (0.01) μm^2^/ms	0.16 (0.03) μm^2^/ms	<0.001
〈MD〉	0.19 (0.03) μm^2^/ms	0.20 (0.03) μm^2^/ms	0.59
*FA*	0.78 (0.04)	0.36 (0.12)	<0.001
*θ*	31 (2) deg	46 (3) deg	<0.001

**FIGURE 5:**
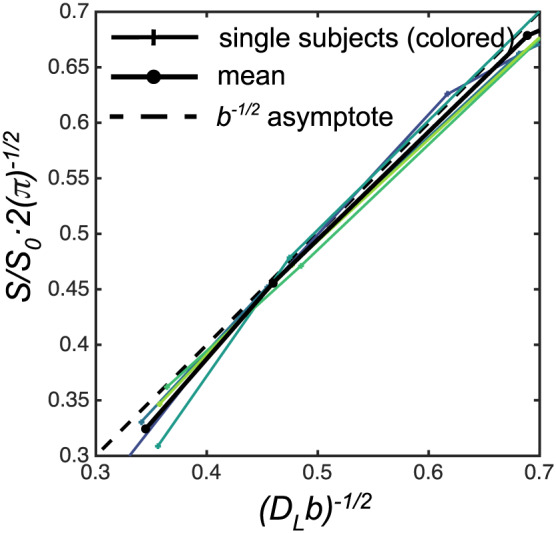
The asymptotic behavior of a powder‐averaged signal from a system described by sticks follows *b* ^−1/2^ at high *b* (low *b* ^−1/2^). Data from the five subjects with voxels in PWM are shown for tNAA. All lines are dimensionless and scaled with their individually fitted *S*_0_ and *D*_L_(sticks)

Figure 5 shows the asymptotic behavior of the attenuation curves where a signal described by the stick model (Equation 2) approach is linear for *b*^−1/2^ → 0 with an intercept at the origin indicated by the dotted line in the plots. This linear slope deviates by less than 0.5% from the stick model for (*D*_L_*b*)^−1/2^ < 0.5. This range is not densely sampled in our data but is covered by at least two *b*‐values in all acquisitions (individual scaling depends on the variation in the fitted *D*_L_). We note that the attenuation on average is stronger than *b*^−1/2^ (below the asymptote), but the deviation is of the order of the noise figures in the measurement.

### 3.2 Simulations

**FIGURE 6:**
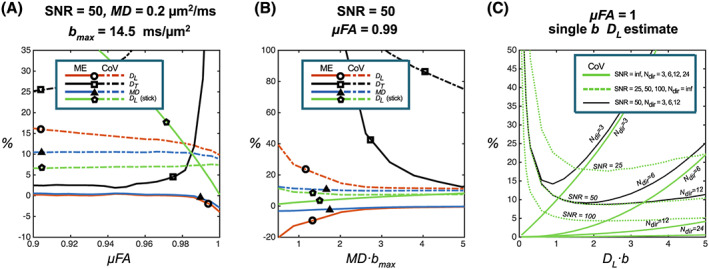
The noise propagation and the effect of acquisition parameter choice in model estimates (ME and CoV) from powder‐averaged data. A (Simulation (i)), Varying degrees of the underlying ground truth microscopic anisotropy with a fixed MD estimate from a set of five *b*‐values comparable to the PWM acquisition. B (Simulation (i)), ME and CoV of estimates with varying MD and a fixed μFA = 0.99. A and B consider the effect of a noise level of SNR = 50 for perfectly powder‐averaged signals described by Equation 1. C (Simulation (ii)), CoV of *D*_L_(sticks) estimates over 1024 orientations of one underlying stick substrate from a single nonzero *b*‐value for an increasing number of gradient directions under noise‐free conditions (solid lines, *N*_dir_ = 3, 6, 12, and 24 with increasing precision). ME converges to zero for the average over uniform substrate orientations. Dotted lines, the influence of noise for a perfect powder average (Simulation (ii)) in a single *b*‐value estimate (*N*_dir_ = inf., SNR = 25, 50, and 100). The black lines indicate the combined effect of SNR = 50 and *N*_dir_ = 3, 6, and 12 (Simulations (i) and (ii) combined). The stated SNRs reflect the noise levels at *b* = 0

**FIGURE 7:**
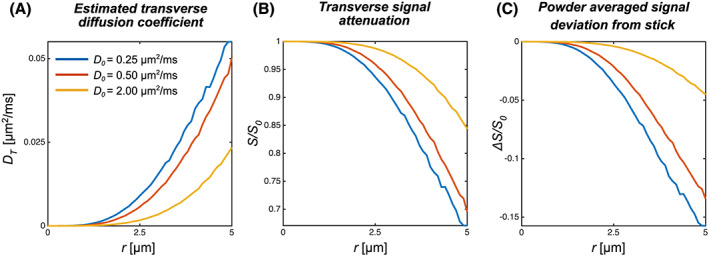
Monte Carlo simulations (Simulation (iii)) illustrating the interpretation of transverse diffusivity as a cylinder radius. A, Estimated *D*_T_ as a function of radius for varying *D*_0_. B, The normalized signal under ideal conditions with a gradient direction perpendicular to the cylinder axis as a function of cylinder radius for the highest *b*‐value (14.5 ms/μm^2^) with varying *D*_0_. C, Deviation from the powder‐averaged signal of a stick (*r* = 0) for systems with the same MD at the same diffusion weighting as in B

The simulations for noise propagation (Simulation (i) in Section 2.1.4) are presented in Figure 6A and 6B, results for the number of gradient directions (Simulation (ii)) and combined effects with noise (Simulation (i)) are shown in Figure 6C, and Monte Carlo simulations of restriction size (Simulation (iii)) are shown in Figure 7. Figure 6A shows parameter estimates with varying ground truth μFA, using *b*‐values equivalent to the PWM acquisition and a similar SNR = 50 and MD fixed at a value in the range of tNAA in WM. We observe minor biases (ME <1%) in MD and *D*_L_ estimates, and the CoVs are of the order of 10% from powder averages over the subdomains with different μFAs. Larger ME and CoV are however found for *D*_T_ and in particular for *D*_T_ → 0 and μFA ~ 1. Simulations with increasing maximum *b*‐value and fixed ground truth μFA and MD are shown in Figure 6B. Here, estimates of MD and *D*_L_ stabilize at (MD *b*_max_) ~ 2. Larger values are however required to capture *D*_T_, but it is, in general, greatly overestimated. Another simplified approach is shown in Figure 6C. Given a negligible *D*_T_, we could fit the stick model from a shorter acquisition with powder averages of only two measurements, eg *b* = 0 and one nonzero *b*‐value. The simulations using this ground truth confirm the lowest CoV around the optimal *bD*_L_~2.285 shown at different noise levels. At an experimentally feasible SNR = 50 a CoV less than 10% is achievable. A substrate with a large alignment and high anisotropy, such as the CC, may induce an additional rotational variance concerning its orientation relative to the gradient vectors even under noise‐free conditions. This variance increases with increasing *b*‐values, but a larger number of directions decreases this bias, as illustrated by Figure 6C. The 12 directions used here account for a CoV less than 1% at an optimal *b*‐value. The combined effect of the noise and a limited number of gradient directions is shown as black curves in Figure 6C. This result indicates that the powder averages with a low number of gradient directions generally benefit from *bD*_L_ < 2.285. For three orthogonal directions an optimal nonzero *b‐*value is found at *bD*_L_~1.

The results from the Monte Carlo simulations in cylindrical restrictions with the gradient waveforms used in the experiments are shown in Figure 7 for different free diffusivities in the range of our fitted values of *D*_T,_ a plausible value for the intra‐axonal free diffusivity *D*_0_ = *D*_L_ of water (2 μm^2^/ms),^34^ tNAA (0.5 μm^2^/ms from this study), and a hypothetical molecule with even lower diffusivity (0.25 μm^2^/ms). Higher apparent *D*_T_ relates to a larger axonal radius or lower free diffusivity, as shown in Figure 7A. The maximum signal attenuation perpendicular to the cylinder axis at the maximum *b*‐value used is shown in Figure 7B and the divergence from the stick signal in Equation 2 is shown in Figure 7C.

## 4 DISCUSSION

The morphological and physiological properties of cells are often modulated by disease: structural damage and protein aggregation can affect the intraneuronal diffusion properties in diseases such as multiple sclerosis and Alzheimer's disease. In this context, the metabolite diffusion measured with magnetic resonance techniques gives a unique window into cell‐specific morphology and physiology in the study of healthy and pathological tissue. In this work, we presented powder‐averaging as an efficient way to collect and analyze DW‐MRS data across tissue with an unknown principal direction and different degrees of macroscopic fiber dispersion over typically large voxel sizes. While DWI data exhibit potential sources of nonmonoexponential decay beside dispersion, eg from water in intra‐ versus extracellular spaces,^40^ DW‐MRS measurements may reflect individual types of intracellular geometry. We acquired the DW‐MRS data by combining a large number of gradient directions (*N* = 12) and high diffusion weighting (up to 14.5 ms/μm^2^), compared with earlier human studies.^22^, ^26^, ^35^ In the CC, we found a large variation in signals across different directions compared with the PWM, reflecting a high alignment of axons (Figure 3). The macroscopic anisotropy of tNAA diffusion was less pronounced in PWM, which also captures more crossing fiber regions. The powder‐averaged signals were comparable in the two regions, suggesting that this procedure discards residual alignment on a voxel level, even when the VOI is placed on a highly organized white matter tract. We considered models interpreting the nonmonoexponential signal attenuation as multiexponential, given by a uniform orientational distribution of local cylindrically symmetric diffusion tensors. This situation allowed for the estimation of the transverse and longitudinal diffusivities *D*_L_ and *D*_T_ and their derived MD and μFA. No substantial difference between the microscopic neuronal morphology was expected between CC and PWM, and accordingly comparable values were found across the two regions. Further, the derived MD from the powder averages and the 〈MD〉 of the macroscopic diffusion tensor were also in good agreement.

The anisotropic diffusion and apparent diffusion coefficients based on water diffusion quantified with DTI‐derived metrics provide sensitive markers for changes in white matter^1^, ^38^ and can also be applied in DW‐MRS.^24^, ^41^ As an important motivation for our approach, the interpretation of FA, and hence of FA differences caused eg by disease, is ambiguous because both macroscopic dispersion and changes to the individual cells on a microscopic level contribute to the anisotropy measured over a voxel. The FA is, therefore, typically a more appropriate biomarker for cellular changes in regions with a high degree of axonal alignment, but entangles microscopic and macroscopic information. The sensitivity is lowered in regions with high dispersion where FA approaches zero. Selectively lowered μFA in one out of many crossing fiber populations may even lead to an increased FA at a voxel level.^42^ Dispersion is a particularly acute problem in more heterogeneous tissues, most notably in cortical gray matter, where the macroscopic FA is negligible, but μFA, in contrast, can retrieve information regarding cell structure.^43^ Dispersed anisotropic microscopic domains also introduce nonmonoexponential signal attenuation that biases diffusion tensor estimation unless the initial slope is well characterized by also estimating higher‐order terms to sufficient degree from multiple *b*‐values.^3^, ^44^ This effect should therefore also be considered as done in our fits when comparing tensor metrics across regions with different degrees of macroscopic fiber dispersion. The ambiguities in interpretation from dispersion and potential misestimation from nonmonoexponential attenuation are expected to be more severe in DW‐MRS data, where large voxel sizes are needed for sufficient SNR. The high μFA ~ 1 found in both regions in our data suggests the main contribution from very thin fibrous cell shapes. Earlier studies estimating tNAA *D*_L_ with the same approach (but with low gradient angular resolution) in rodents report slightly lower values (0.36 and 0.33 μm^2^/ms),^22^, ^23^ which could be explained by substantial differences between rodent gray matter and human white matter. However, considerably larger values similar to ours were found by Shemesh et al (*D*_L_ = 0.51 μm^2^/ms), although this may be explained by a different experimental setting and analysis approach.^45^ Comparisons can also be made with MD from earlier diffusion tensor DW‐MRS in humans.^28^, ^46^, ^47^ Neglecting the effects of nonmonoexponential attenuation and assuming the stick model, those findings translate to 3 MD = *D*_L_ = 0.63–0.78 μm^2^/ms, which is close to our values (0.65 μm^2^/ms (CC) and 0.58 μm^2^/ms (PWM)). These studies also report values of tNAA FA below 0.6 even in highly aligned structures such as the CC, which is likely to be significantly affected by dispersion of fibers over the voxel volume. Our values are also considerably higher than μFA estimates from water diffusion using nonconventional DWI, where extracellular water may introduce a component with lower anisotropy.^48^ The ratio between *D*_L_ and the free diffusivity at body temperature (*D*_0_) reflects the tortuosity and viscosity of the intra‐axonal space, and is for water typically reported as ~2/3 = 0.66.^49^ The free diffusivity of NAA at room temperature has been reported to be 0.75 μm^2^/ms on phantoms with a similar measurement setup on a human scanner.^25^ At body temperature a similar proportional increase as for water (2.0 μm^2^/ms and 3.0 μm^2^/ms at room and body temperatures) could roughly be expected from the Stokes‐Einstein relation and would translate to about 1.1 μm^2^/ms (not taking eg temperature and compound‐dependent changes in effective hydrated radius into account). This gives us *D*_L_/*D*_0_ ratios of 0.52 (PWM) and 0.58 (CC), which are lower than that of water. However, Kroenke et al^22^ reported a free diffusion coefficient for NAA of 0.78 μm^2^/ms in a phantom at body temperature in an NMR setup, giving ratios of 0.74 (PWM) and 0.83 (CC) from our data. Differences in reported free diffusivities may originate from differences in measurement methods or phantom composition, which may influence the diffusivities of individual metabolites.^25^ Deviations in *D*_L_/*D*_0_ compared with water could reflect another compartmentalization or measurements on shorter length‐scales compared with the tortuosity of the intra‐axonal space. This has microstructural information potentially complementary to that accessible through modelling of water diffusion data.

Transverse diffusivity can be related to axonal diameter under the assumption of simple geometries, and has been widely used to model both DW‐MRS and DWI data.^50^, ^51^ Our Monte Carlo simulations were performed to illustrate the interpretation of a nonzero transverse diffusivity, the related signal attenuations and its derived *D*_T_. The axon radius in the CC is expected to be below *r* = 1.13 μm for 98% of a volume‐weighted distribution.^28^, ^52^ The contribution to the signal attenuation of such small restrictions with the gradient settings used here is well below 1% and the noise levels that are realistic for human DW‐MRS data (Figure 7B and 7C), which has also been pointed out in other simulation studies.^53^, ^54^, ^55^ Likewise, DW‐MRS data acquired from the mouse brain with *b*‐values up to 60 ms/μm^2^ suggest that axonal radii estimated from tNAA diffusion are negligible.^23^ Model fits in PWM with higher SNR levels and *b*‐values compared with CC show little evidence for a substantial effect of a nonzero *D*_T_ (comparing solid gray and dashed gray lines in the top row of Figure 4). This is further shown in Figure 5,where we considered an alternative way to investigate the influence of transverse diffusivity, considering the functional form of the attenuation curves at high *b*‐values.^34^ The benefit of this approach is that it discards the fast‐diffusing, heterogeneous signal contributions from the extracellular space in the case of water measurements, as well as large spherical restrictions (eg somas) that may be difficult to model and have not been observed so far in DW‐MRS studies.^56^, ^57^ Human white matter water DWI data and a post hoc analysis of the NAA DW‐MRS rat data from Kroenke et al^22^ follow a *b* ^−1/2^‐scaling compatible with the stick model.^3^, ^34^ We obtained similar results, shown in Figure 5. Group averaged data deflects below the asymptote with a slightly faster decay than described by Equation 2, in line with a nonzero transverse diffusivity or residual isotropic components, such as that originating from cell somas and small‐fiber undulations or exchange between branches of fibers with different directions below the characteristic length scales of the diffusion process.^19^, ^58^, ^59^ This deviation is however not apparent in single‐subject data and on the order of the noise levels. With only two *b*‐values on the apparent asymptotic part of the curve, the scaling coefficient could not be verified either. Future experiments should consider a wider range of *b*‐values in the high range to estimate the scaling behavior to conclude on those observations.

Our simulations of noise propagation indicate that a high true μFA leads to highly overestimated *D*_T_‐values with large CoV and associated underestimation of the μFA (Figure 6A). Sufficient signal attenuation related to (*b* MD) in Figure 6B is needed to capture the signature of a low but nonzero *D*_T_, which is otherwise overestimated. To compare with our experimental data, (*b* MD)~2 and 3 for tNAA in CC and PWM respectively.

Data shown here can help design DW‐MRS protocols for human applications requiring shorter scan times than those used in this study. Our findings suggest that the nonmonoexponential attenuation of at least tNAA in white matter with current gradient limitations is well captured by the simple stick model. In other words, *D*_L_ contains the relevant representation of the axonal microstructure while large biases and CoV in *D*_T_ estimates provide little additional information. This was however less evident in CC, which may be explained by the lower SNR and lower maximum *b*‐value compared with the PWM data. We also note that our estimates of tNAA *D*_L_ are in good agreement with earlier model‐based analyses of both human and animal data.^17^, ^45^ Two considerations could guide the implementation of an optimized protocol given this simplification. First, a sufficient number of uniformly oriented gradient directions should be applied to provide a rotationally invariant powder average, which is important for providing comparable multiexponential attenuation across measurements with different and varying configurations. The simulated situation in Figure 6C, considering the worst‐case scenario with high fiber alignment, indicates that the 12 directions used in this study relate to a CoV less than 1% in the *D*_L_ estimate for arbitrarily oriented fibers in relation to the gradient orientations and the given range of *b*‐values. A lower number of directions may induce significant rotational variance unless the orientational distribution can be estimated by other means. Note that the same source of rotational variance would similarly affect a simple MD estimate. Second, by fitting only *D*_L_ in Equation 2, only one nonzero *b*‐value is sufficient. The maximum sensitivity in signal for variation in *D*_L_ found from derivation of Equation 2 is found at *bD*_L_ = 2.285, which was also confirmed by the noise propagation simulation in Figure 6C. For tNAA, this translates to *b* ~ 4 ms/μm^2^, which is considerably lower than our setting. The flatness of the noise‐induced CoV with respect to *bD*_L_ suggests that lower *b*‐values and numbers of directions may also be sufficient, depending on whether SNR or rotational variance is the dominating source of CoV. This could be easily realized with the benefits of shorter *T*_E_ and associated SNR increases. As multiple averages in general are needed for DW‐MRS, identifying the minimal required number of experimental parameters has impact for studies on clinical populations or for the additional repetitions needed for imaging.^60^, ^61^, ^62^ While robust *D*_T_ estimation in white matter requires unrealistic SNR and *b‐*values for the current experimental setting, several questions still remain regarding other scenarios where *D*_T_ could be higher. This could be metabolites residing in other cell types (such as tCho and tCr), white matter pathologies, gray matter, or applications to pathologies outside the brain such as muscle or tumor cells with larger diameter. In those situations, a range of *b*‐values would still be informative.

Axonal dispersion has been investigated in earlier studies in human DW‐MRS data from the CC by modeling the orientational distribution in measurements perpendicular and parallel to the main fiber direction.^17^, ^28^ Even though the dispersion of axons per se could contain valuable pathological information,^63^ our powder‐averaging approach circumvents the need for fitting the distribution and does not require prior information regarding the main fiber orientation as in previous DW‐MRS acquisitions and analyses.^17^ Another approach accessible with a larger number of gradient directions is to estimate the orientational dispersion with increasing order of spherical harmonics, which also better conditions the estimation of multiple compartments.^49^ With our 12 directions the spherical harmonics can be expanded to order *l* = 2, which corresponds to the diffusion tensor. On this level and given the assumption of a disperse stick, the two minor eigenvalues of the macroscopic diffusion tensor (given a correct assessment of higher‐order terms) provide a measure of the width of the fiber dispersion around the principal direction in two orthogonal directions.^33^, ^64^ Our estimates of *θ* were as expected lower in CC compared with PWM (mean *θ* = 31° versus 46°). Isotropic diffusivity in terms of the diffusion tensor is equivalent to a spread at the magic angle (~54.6°).

The stick model is limited to situations where the prevalent morphology is that of elongated and very thin fibers and does not account for a finite presence of other geometries, such as those of cell bodies. A more realistic representation of the intracellular space in the brain would require a clear deviation from the stick model,^40^, ^65^ which in turn could provide additional morphological information in regions rich in cell somas, with large degree of more isotropic restricted diffusion, such as that observed in the granular layers of hippocampus and cerebellum.^66^, ^67^ The deliberately simple models used in this study only consider dispersion of identical compartments. As seen in Figure 5, deviations from the model assumptions are consistent but small, and little fitting power is left to isolate the existence of additional compartments conclusively and from there characterize and quantify the contributions of these compartments to the signal. The problem of multiple model interpretations describing the same diffusion‐encoded data may also call for more elaborate diffusion‐encoding schemes isolating more specific signatures of the individual subvoxel diffusion processes. An approach to enhance the specificity to particular compartment shapes of recent interest in the DWI community is the use of double diffusion encoding (DDE) or multidimensional diffusion encoding techniques.^68^, ^69^ An important aspect of this approach is that it disentangles the contributions to the multiexponential attenuation related to variation in mean diffusivities not captured by the conventionally encoded data used in this study. The combination of DDE and DW‐MRS has been applied in preclinical settings,^15^, ^45^, ^70^ and we recently demonstrated its feasibility in a human setup where high tNAA μFA values were also observed.^71^, ^72^ A wider range of diffusion times could also be an additional handle to separate exchange processes across compartments or isolate restriction sizes.^73^ Examples from water diffusion measurements probing short diffusion times with oscillating gradient spin echo methods or other approaches modulating the spectral content of encoding waveforms demonstrate the large effects in cell body rich domains such as the granular layer of the cerebellum^66^, ^67^, ^74^ and similar approaches have also been used for DW‐MRS.^57^, ^75^ From an intra‐axonal perspective, measurable effects of decreased water *D*_L_ at longer diffusion times (50‐600 ms) may stem from variations in axonal radius,^76^ which could also be an interesting effect to probe with NAA. Figure 7 illustrates another benefit of using metabolite diffusion as a structural probe compared with water measurements: the lower free diffusion coefficients of metabolites relate to larger signal attenuations in small restrictions, making them more sensitive geometrical probes than water, given the same gradient hardware constraints. This seemingly counterintuitive effect can be understood by considering the larger displacement of the smaller encoding center of the mass propagator given by lower diffusivities.^77^ While the proposed models reflecting multi‐Gaussian situations are close fits to data, additional complexity might be constituted by non‐Gaussian behavior within the individual compartments. This could for instance be due to cytoplasmatic substructures or branching and undulating fibers represented by subdiffusion or more complex geometrical models, as recently proposed.^26^, ^58^

There are some limitations to this study. Already mentioned is the possible contribution of geometries other than disperse fibers to the nonmonoexponential attenuation, calling for alternative diffusion‐encoding approaches. Systematic biases and noise sources could be induced by improper phasing and additional attenuation from eg physiological motion more prone to affect higher *b*‐values or by magnetization transfer affecting metabolites coupled to the non‐completely‐suppressed water resonance. The subject motion could also induce additional variance across repetitions, and this motion could be improved with prospective motion correction techniques.^78^, ^79^ The PRESS localization results in significant chemical shift displacements, which in our study restricted reliable quantification to tNAA only, particularly in the CC. This circumstance could be improved with LASER‐based DW‐MRS techniques,^6^ which will be addressed in future studies. The additional effect of cross terms from localization gradients may result in slight deviations in the diffusion weightings of different gradient directions, which challenge the necessary requirement for a powder average of uniform gradient directions with *b*‐values on unique shells. This potential bias was investigated numerically in the supplementary material and was found to be low in our settings. However, it should be considered in other sequences and could be partially reduced by omitting crusher gradients and *b* = 0 measurements or by adjusting the diffusion gradient vectors and amplitudes.^80^, ^81^ A larger number and higher maximum diffusion weightings would also better resolve the long tail of the diffusion weighting to possibly detect additional substructures such as cell bodies or even organelles. The limited gradient strength of the scanner used, and SNR, pose a limit to the maximum possible *b*‐value in the current setting. Future experiments could take advantage of recent improvements in gradient hardware in human scanners to further improve future powder‐averaged DW‐MRS acquisitions.^82^

## 5 CONCLUSION

The measurement of metabolite diffusion provides useful cell‐specific information, but the macroscopic arrangement within large voxel sizes must be considered for unbiased microstructural interpretations. Here we demonstrate how powder averaging can be used to handle angular dispersion effects in the acquisition and analysis of DW‐MRS data. Noise propagation considerations and data suggest that the nonmonoexponential attenuation in human white matter of tNAA up to *b*‐values of 10 000‐14 000 s/mm^2^ is well described by a model comprised of thin cylinders characterized by a single longitudinal diffusivity *D*_L_. This insight provides useful input to future simplified protocols using DW‐MRS as a cell‐specific biomarker. The possibility of measuring *D*_L_(tNAA) in any white matter region has a high clinical value as a specific marker for axonal health. Future powder‐averaged measurements from creatine and choline compounds may give further specificity to morphological changes from glial reactivity in neuroinflammation.

**Abbreviations**
*b*, diffusion‐weighting *b*‐value
*b*_max_, maximum *b*‐value
CC, corpus callosum
CoV, coefficient of variation
CRLB, Cramér‐Rao lower bound
〈**D**〉, voxel average diffusion tensor
*D*_0_, free diffusivity
DDE, double diffusion encoding
*D*_L_, micro domain longitudinal diffusivity
〈*D*_L_〉, voxel average longitudinal diffusivity
*D*_T_, micro domain transverse diffusivity
〈*D*_T_〉, voxel average transverse diffusivity
DWI, diffusion‐weighted imaging
DW‐MRS, diffusion‐weighted MRS
FA, fractional anisotropy
MD, mean diffusivity
ME, mean error
NAA, *N*‐acetyl aspartate
*N*_dir_, number of gradient directions
PRESS, point‐resolved spectroscopy
PWM, parietal white matter
SNR, signal to noise ratio
VOI, volume of interest
*δ*, gradient duration
*Δ*, gradient separation
*θ*, angular dispersion
μFA, microscopic fractional anisotropy
*τ*, bipolar delay.

## Supporting information

Figure S1: Simulations considering the additional effects of diffusion weighting of localization and crusher gradients of the bipolar PRESS DW‐MRS sequence. A) PWM acquisition, B) CC acquisition. Left panels: Calculation of b‐value with diffusion gradient only (b_diff_, x‐axis) vs. the deviation when including crusher and slice gradients (Delta b, y‐axis). The full B‐matrix was calculated for the system including cross‐terms. b‐value with diffusion gradients turned off was 0.0032 ms/μm^2^ and the difference between intended and actual weighting ranged between +/− 0.065 ms/μm^2^ at b_diff_ = 14.5 ms/μm^2^. The different lines show the deviation for the 12 different diffusion gradient directions. Right panels: Attenuation curve for theoretical powder average equation 2 for a stick with DL = 0.5 ms/μm^2^ (red curve) and powder averages calculated from the full B‐matrix calculation including gross terms (dashed blue and solid black curves). The powder average of a single direction from 12 gradient directions will depend on its relative orientation to the substrate. Standard deviation over 1000 uniform orientations is plotted as thin black lines and span +/− 1.5% of the signal at maximum diffusion weighting. For a substrate with full dispersion this difference is less than 0.1% (dashed blue line).
