# Supplementary material for: Cytosolic diffusivity and microscopic anisotropy of N‐acetyl aspartate in human white matter with diffusion‐weighted MRS at 7 T
Source: NMR Biomed. 2020 Mar 31;34(5):e4304. doi: 10.1002/nbm.4304 (PMC8244075; doi:10.1002/nbm.4304)
Supplement: Supplementary file 1 — Figure S1: Simulations considering the additional effects of diffusion weighting of localization and crusher gradients of the bipolar PRESS DW‐MRS sequence. A) PWM acquisition, B) CC acquisition. Left panels: Calculation of b‐value with diffusion gradient only (bdiff, x‐axis) vs. the deviation when including crusher and slice gradients (Delta b, y‐axis). The full B‐matrix was calculated for the system including cross‐terms. b‐value with diffusion gradients turned off was 0.0032 ms/μm2 and the difference between intended and actual weighting ranged between +/− 0.065 ms/μm2 at bdiff = 14.5 ms/μm2. The different lines show the deviation for the 12 different diffusion gradient directions. Right panels: Attenuation curve for theoretical powder average equation 2 for a stick with DL = 0.5 ms/μm2 (red curve) and powder averages calculated from the full B‐matrix calculation including gross terms (dashed blue and solid black curves). The powder average of a single direction from 12 gradient directions will depend on its relative orientation to the substrate. Standard deviation over 1000 uniform orientations is plotted as thin black lines and span +/− 1.5% of the signal at maximum diffusion weighting. For a substrate with full dispersion this difference is less than 0.1% (dashed blue line). [file NBM-34-e4304-s001.docx]

Supplementary material

**Cytosolic diffusivity and microscopic anisotropy of N-acetyl aspartate in human white matter with diffusion weighted magnetic resonance spectroscopy at 7 tesla**

Authors: Henrik Lundell^1*^, Carson Ingo^2,3^, Tim Dyrby^1,4^, Itamar Ronen^5^

1. Danish Research Centre for Magnetic Resonance, Centre for Functional and Diagnostic Imaging and Research, Copenhagen University Hospital Hvidovre, Denmark
2. Department of Physical Therapy and Human Movement Sciences, Northwestern University, Chicago, IL, USA
3. Department of Neurology, Northwestern University, Chicago, IL, USA
4. Department of Applied Mathematics and Computer Science, Technical University of Denmark, Kongens Lyngby, Denmark
5. C. J. Gorter Center for High Field MRI, Department of Radiology, Leiden University Medical Center, Leiden, The Netherlands

*Corresponding author:

Henrik Lundell

Address:

Danish Research Centre for Magnetic Resonance

Copenhagen University Hospital Hvidovre

Kettegaards Allé 30

3480 Hvidovre

Denmark

E-mail: [lundell@drcmr.dk](mailto:lundell@drcmr.dk)

Phone: +45 38620505

Supplementary figure S1: Simulations considering the additional effects of diffusion weighting of localization and crusher gradients of the bipolar PRESS DW-MRS sequence. A) PWM acquisition, B) CC acquisition. Left panels: Calculation of b-value with diffusion gradient only (b_diff_, x-axis) vs. the deviation when including crusher and slice gradients (Delta b, y-axis). The full B-matrix was calculated for the system including cross-terms. b-value with diffusion gradients turned off was 0.0032 ms/µm^2^ and the difference between intended and actual weighting ranged between +/- 0.065 ms/µm^2^ at b_diff_ = 14.5 ms/µm^2^. The different lines show the deviation for the 12 different diffusion gradient directions. Right panels: Attenuation curve for theoretical powder average eq. 2 for a stick with DL = 0.5 ms/µm^2^ (red curve) and powder averages calculated from the full B-matrix calculation including gross terms (dashed blue and solid black curves). The powder average of a single direction from 12 gradient directions will depend on its relative orientation to the substrate. Standard deviation over 1000 uniform orientations is plotted as thin black lines and span +/- 1.5 % of the signal at maximum diffusion weighting. For a substrate with full dispersion this difference is less than 0.1% (dashed blue line).
